# Longitudinal Single Photon Emission Computed Tomography Neuroimaging as an Indication of Improvement in Psychiatric Disorders in a Community Psychiatric Practice

**DOI:** 10.3389/fpsyt.2022.787186

**Published:** 2022-03-25

**Authors:** John F. Thornton, Howard Schneider, Philip F. Cohen, Simon DeBruin, John Michael Uszler, Yin-Hui Siow, Mary K. McLean, Muriel J. van Lierop, Dan G. Pavel, Theodore A. Henderson

**Affiliations:** ^1^Rossiter-Thornton Associates, Toronto, ON, Canada; ^2^International Society of Applied Neuroimaging, Denver, CO, United States; ^3^Sheppard Clinic North, Vaughan, ON, Canada; ^4^Nuclear Medicine, Lions Gate Hospital, Vancouver, BC, Canada; ^5^Department of Radiology, University of British Columbia, Vancouver, BC, Canada; ^6^Good Lion Imaging LLC, Columbia, MD, United States; ^7^DrSPECTscan Inc., Lake Elsinore, CA, United States; ^8^Department of Molecular and Medical Pharmacology, University of California, Los Angeles, Los Angeles, CA, United States; ^9^Nuclear Medicine, Southlake Regional Health Centre, Newmarket, ON, Canada; ^10^Private Practice, Toronto, ON, Canada; ^11^PathFinder Brain SPECT LLC, Deerfield, IL, United States; ^12^Dr. Theodore Henderson, Inc., Denver, CO, United States; ^13^The Synaptic Space, Inc., Denver, CO, United States; ^14^Neuro-Luminance, Inc., Denver, CO, United States

**Keywords:** neuroimaging, SPECT, biomarker, traumatic brain injury, attention-deficit hyperactivity disorder, post-viral syndrome, community psychiatry

## Abstract

In the community, there is a need to more objectively evaluate the response of common chronic psychiatric disorders to treatment. Brain single photon emission computed tomography (SPECT) indirectly measures cerebral functional activity by uptake of a radiotracer, which follows regional cerebral blood flow. Brain 3D Thresholded SPECT scans are thresholded three dimensional images derived from brain SPECT data. A retrospective community study of longitudinal (before and after treatment) brain 3D Thresholded SPECT scans of 73 patients with all-cause psychiatric disorders (most frequent diagnostic clusters: attention-deficit hyperactivity disorder, post-mild traumatic brain injury, affective disorders, psychotic disorders, post-viral chronic syndromes), shows these baseline SPECT scans predict improvement (non-worsening to large improvement) in clinical functioning with a sensitivity of 94% (95% confidence interval 86–98%) and a specificity of 67% (95% confidence interval 21–94%). In contrast, contemporaneous analysis by the same radiologist of conventional 2D reading of the same before and after treatment brain SPECT scan data of the same 73 patients, predicted improvement (non-worsening to large improvement) in clinical functioning with a sensitivity of only 26% (95% confidence interval 17–37%) although with a specificity of 100% (95% confidence interval 44–100%). These data suggest 3D Thresholded SPECT scans can provide the clinician with a more objective measure for verifying improvement in psychiatric disorders seen in the community, consistent with prior studies of SPECT as a measure of neurobiological change. Furthermore, these data suggest 3D Thresholded SPECT scans may have clinical application in guiding treatment and potentially improving outcomes.

## Introduction

There is a need in community-based psychiatric practice to more accurately evaluate the response of common chronic psychiatric disorders to treatment. While the clinical exam, as well as various functional questionnaires, can and should continue to be used in the evaluation of a patient’s progress, more objective tests are needed to directly evaluate the patient in an objective manner. At the time of this writing, there is still a paucity of clinically available and useful biomarkers in the field of psychiatry (1). Regardless of diagnosis and independent of the Diagnostic and Statistical Manual (DSM)-IV, DSM-IV-TR, or DSM-V criteria, symptoms such as pain, apprehension, distress, inflexibility, and/or cognitive clarity are important markers of treatment progress which remain largely subjective. The lack of psychiatric biomarkers impedes the clinician’s ability to deliver the best personalized care.

Perfusion single photon emission computed tomography (SPECT) brain scans can measure aspects of brain function. The amount of radiotracer uptake in a brain region is proportional to the blood flow within gray matter over the physiological range. Since local cerebral blood flow is proportional to neurophysiological function, perfusion SPECT provides a one-off measure of brain function (2–4). Further computer processing of the SPECT data can result in a three-dimensional looking image of the brain (termed a brain 3D Thresholded SPECT scan) which provides more clinically relevant information than a two-dimensional tomogram (5).

Brain SPECT perfusion scans are emerging as potential biomarkers for identifying and separating comorbid conditions. For example, perfusion SPECT scans were able to differentiate traumatic brain injury (TBI) and post-traumatic stress disorder (PTSD) with a sensitivity of 92% and a specificity of 85% in a study of 196 veterans (6). Furthermore, these results were replicated in a civilian sample of over 24,000 individuals (7). In a recent study (8) SPECT perfusion scans could differentiate adults with attention-deficit hyperactivity disorder (ADHD) from normal controls with a sensitivity of 100% and a specificity of 97% in a study of over 1,000 individuals using regions of interest analysis of 3D Thresholded scan data. We (HS, JT, MM, and MvL) previously showed that 3D Thresholded SPECT scan analysis yielded greater sensitivity in detecting ADHD compared to conventional 2-D tomographic interpretation in a large retrospective analysis of 427 patients (5). Recently published procedure guidelines by Cohen et al. (9) reviews indications and applications for brain SPECT scans in a range of neuropsychiatric and psychiatric disorders. Pavel et al. (10) recently published an encyclopedic review of the SPECT findings associated with TBI, stroke, PTSD, dementia, and several neuropsychiatric conditions.

Perfusion SPECT has shown promise as a biomarker of previously subjective symptoms. SPECT was recently shown to provide an objective measure of pain in burn patients (11). SPECT also demonstrated specific findings in PTSD distinct from a trauma-exposed cohort (12). Perfusion SPECT has also been shown to provide objective evidence of neurophysiological changes in response to specific treatments, such as multi-watt infrared laser therapy for TBI (13), stem cell therapy for Lyme disease (14), and combined ketamine-transcranial magnetic stimulation (15). Studies have suggested SPECT scan data may improve clinical outcome by guiding treatment choices more quickly to successful strategies (4, 16–18). However, there has not been a study of perfusion SPECT scans as an objective measure of change in the broad spectrum of psychiatric disorders encountered in a community psychiatry practice.

Given the potential of brain SPECT perfusion scans to reveal comorbidities and to objectively indicate improvement in neuropsychiatric functioning, we have implemented the use of such scans in a community psychiatric practice from 2005 to 2019 to provide an additional measure of patients’ clinical improvement, predominately in complex or treatment-unresponsive cases. In this paper we present a naturalistic retrospective study in which we compare the changes in the patients’ 3D Thresholded SPECT scans with the improvements as assessed by conventional clinical observation and interview.

## Materials and Methods

3D Thresholded SPECT brain scans were offered to patients in the community psychiatric practice of one of the authors (JT) when a patient had refractory symptoms that had not responded to conventional treatments at least 6 months after the development of the mental health disorder. Otherwise, no other selection criteria were used. Where 3D Thresholded SPECT scans were initially offered, almost all patients (greater than 95%) accepted them. Risks, such as radiation exposure and the complex nature of the results, were explained to all patients offered scans and informed consent was obtained. All scans of these patients were processed to provide both 3D Thresholded SPECT scan displays and conventional SPECT scan tomographic images from the same raw SPECT data.

A total of 436 patients accepted 3D Thresholded SPECT scans before treatment. These patients were all offered a second scan after a period of treatment, again with informed consent, this time concerning a second scan. 73 of these patients accepted and underwent valid 3D Thresholded SPECT scans on average 450 days after (or during) a treatment. We herein present these 73 patients who received 3D Thresholded SPECT scans before and after (or during) a period of treatment.

Perfusion SPECT scans were performed at one of two tertiary care hospitals in Toronto, Ontario. The scanning procedures followed the guidelines of the Society of Nuclear Medicine (19). An intravenous dosage of 10–20 millicuries of Tc99m-hexamethylpropylene-amine oxime (HMPAO) (*n* = 53) or Tc99m-ethyl cysteinate dimer (ECD) (*n* = 19) was given. The patient then waited 30–60 min, and then the patient’s head was imaged by gamma photon cameras. Both Tc99m-HMPAO and Tc99m-ECD radiotracers are taken up similarly by the brain, although they have small differences in retention and imaging activity (20). Whether a patient received Tc99m-HMPAO or Tc99m-ECD was independent of clinical diagnosis or other patient factors and depended on supply issues. In all cases except for one, a patient received the same type of radiotracer for the first and second SPECT scans.

**FIGURE 1:**
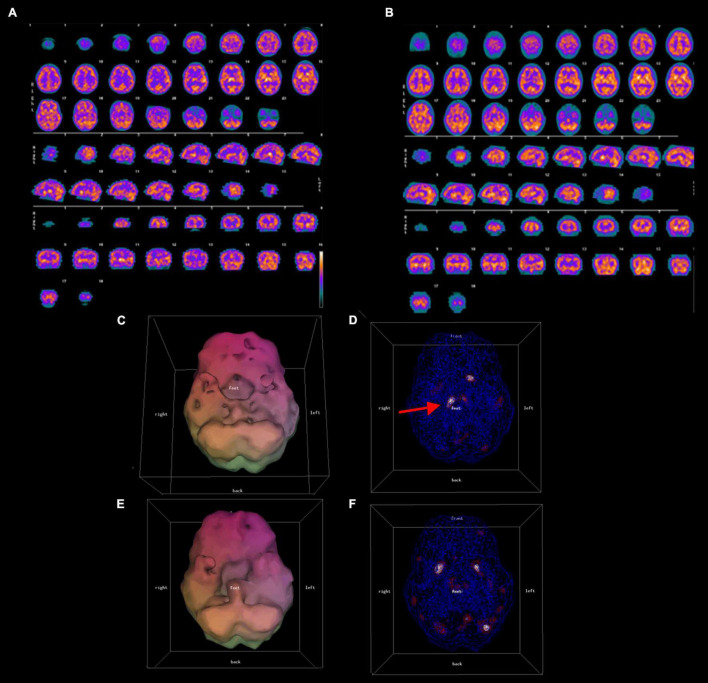
Conventional **(A,B)** and 3D thresholded **(C–F)** displays of pre-treatment **(A,C,D)**, and post-treatment **(B,E,F)** SPECT scan results for one representative patient. A 49 year old male with symptoms of ADHD and mood dysregulation underwent a SPECT scan. Both conventional **(A)** and 3-D thresholded SPECT displays **(C,D)** prior to treatment show diffuse cortical hypoperfusion most severe in the bilateral temporal lobes and the orbitofrontal cortices, as well as over-activity of the thalamus [red arrow in panel **(D)**]. Post-treatment scans **(B,E,F)** show improved temporal, dorsal frontal, and parietal lobe perfusion, normalization of thalamic perfusion, and a reduction of hypoperfusion in the orbitofrontal cortices.

**FIGURE 2:**
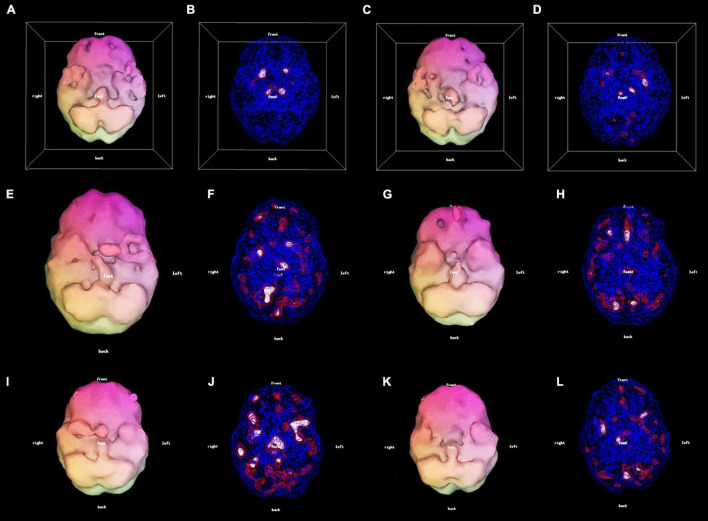
3D thresholded displays: 56-year-old female with Lyme disease pre-treatment **(A,B)** and post-treatment **(C,D)** clinically improved. 33-year-old male with mood dysregulation and ADHD pre-treatment **(E,F)** and post-treatment **(G,H)**. There was clinical improvement with a mood stabilizer. However, due to the prefrontal hypoperfusion **(G)** a stimulant was added resulting in further improvement. 26-year-old female with mTBI pre-treatment **(I,J)** and post-treatment **(K,L)** clinically improved.

All patients were scanned with a Picker Prism 3000 three-headed camera with continuous acquisition of a 128 × 128 pixel image via 120 steps (3 degrees/step) using fan beam collimation. A cloud of data pixels was obtained which represented the photons registered from the patient’s brain. The cloud of data pixels was corrected for attenuation and filtered. The cloud of processed pixels was then sliced into orthogonal tomographic planes to produce what Schneider et al. (5) define as the “conventional SPECT” scan. As well, the cloud of pixels was also thresholded at various levels, i.e., only areas of activity exceeding a particular level of activity of the most active area of the patient’s brain were displayed. For example, for surface views of the brain in the 3D Thresholded SPECT scans the pixels were thresholded at 55%, i.e., only pixels representing activity exceeding 55% of the most active areas of the brain were displayed. Within the interior of the brain, pixels were thresholded at levels representing activity exceeding 55, 85, and 92% of the most active areas of the brain. These thresholds reflect the work of Mena et al. (21), Darcourt et al. (22), and Payne et al. (23), and have been used by Amen (24) in tens of thousands of SPECT scans. Additional details concerning the scanning and thresholding process is given by Thornton et al. (25). These thresholded pixels are then rendered into a 3D form to produce what Schneider et al. (5) define as the “3D Thresholded SPECT” scan. Figure 1 shows a conventional set of SPECT tomograms, along with an inferior view and a wire-frame view of the interior of the 3D Thresholded SPECT scan of a patient before and after treatment. Figure 2 shows before and after treatment 3D Thresholded SPECT scans of additional patients.

The 3D Thresholded SPECT scans and the conventional SPECT scans from each patient were interpreted by a nuclear medicine radiologist with limited clinical information provided. To reduce confounding, all the radiologists agreed to read the scan based on what was seen in the image, rather than extrapolating from any clinical diagnoses. Some clinical information is required in all requests for imaging services in the community. Typically, the requisition form stated the patient’s age, sex, occupation, the working diagnosis, and in some cases the patient’s clinical progress. However, to reduce confounding and get the most objective comparison of before and after SPECT scans, the patient’s progress was not emphasized. Again, as noted above, the radiologists reading the SPECT scans attempted to do so based on the imaging findings. The same radiologist would read a patient’s 3D Thresholded SPECT images, and then read the Conventional SPECT images. There were four different radiologists who read different patients’ images, but only one radiologist read any one particular patient’s images, (i.e., there is no comparison possible between two different radiologists for one patient’s images).

The radiologists systematically commented on the 3D Thresholded SPECT scans (i.e., providing their opinion about whether the patient’s scan was worsening or improving). In reading the conventional SPECT scans, the radiologists provided a conventional report focused on any abnormalities in different neuroanatomical areas. The brain SPECT atlas compiled by Amen (24) was used as the reference source for normal versus abnormal SPECT images.

The one treating clinician independently considered whether each patient’s clinical condition was worsening or improving as specified by the clinical notes prepared based on conventional clinical observation and patient interview. From this information, we then compared the direction of the patient’s clinical condition, with the results of both the conventional and the 3D Thresholded SPECT scans to determine if the scan results represented a True Positive, False Negative, False Positive, or True Negative, for that patient. An improved or stable (i.e., non-worsening) clinical state with an improved or stable post-treatment SPECT scan compared to pre-treatment SPECT scan, would be a True positive. A worsened clinical state with a worsening in post-treatment SPECT scan compared to pre-treatment SPECT scan, would be a True Negative. An improved or stable (i.e., non-worsening) clinical state with a worsening in post-treatment SPECT scan compared to pre-treatment SPECT scan, would be a False Negative. A worsened clinical state with an improved or stable post-treatment SPECT scan compared to pre-treatment SPECT scan, would be a False Positive.

The True Positive, False Negative, False Positive, and True Negative values for the 73 patients’ 3D Thresholded SPECT scans and the 73 patients’ conventional SPECT scans were then used, by standard statistical methods, to calculate the sensitivity and the specificity for the 3D Thresholded SPECT scans and for the conventional SPECT scans. Software obtained from the Knowledge Translation Program (26) was used for statistical calculations.

## Results

### Study Patient Population

As noted above, 73 patients received 3D Thresholded brain SPECT scans and conventional brain SPECT scans (both created from the same raw scanning data) before and after (or during) a period of treatment. The average age of patients was 49.6 years old with a standard deviation of 11.8 years. 60.3% of the patients were female. All patients were being treated by the same clinician (JT) at the time of both SPECT scans.

The average duration before and after scans, was 450 days. The DSM-5 diagnoses (27) or DSM-5-related diagnostic clusters of the patients are listed in Table 1. Many patients from early in the time period examined had DSM-IV or DSM-IV-TR diagnoses which were converted to equivalent DSM-5 or DSM-5-related diagnoses in Table 1.

**TABLE 1:** Patient diagnoses.

Diagnostic cluster (DSM-5 diagnosis or chapter)	Number of patients	%	Mean age (standard deviation) (years)	Mean duration between scans (standard deviation)(days)
Attention-deficit/hyperactivity disorder	24	33%	47.5 (9.1)	347 (248)
Post-mTBI (mild traumatic brain injury) (DSM-5 chapter: Neurocognitive Disorders, those disorders “due to traumatic brain injury”)	19	26%	45.4 (12.9)	405 (414)
Affective disorders (DSM-5 chapters: Bipolar and Related Disorders, Depressive Disorders)	11	15%	54.4 (16.9)	702 (1,043)
Psychotic disorders (DSM-5 chapter: Schizophrenia Spectrum and Other Psychotic Disorders)	4	5%	55.5 (7.0)	373 (305)
Post-viral chronic (“Long Haul“) syndrome (DSM-5 chapter: Neurocognitive disorders, those disorders due to post-viral sequalae “due to another medical condition”)	4	5%	51.8 (9.6)	263 (159)
Other neurological (DSM-5 chapter: Neurocognitive Disorders, due to neurological conditions)	4	5%	52.0 (11.2)	503 (396)
Chronic fatigue, fibromyalgia (DSM-5 diagnosis: Somatic Symptom Disorder)	3	4%	55.3 (10.0)	653 (479)
Anxiety disorders (DSM-5 chapter: Anxiety Disorders)	2	3%	51.0 (12.7)	896 (1118)
Chronic pain (DSM-5 diagnosis: Somatic Symptom Disorder, persistent, with predominant pain)	1	1%	42	105
Dementia (DSM-5 chapter: Neurocognitive Disorders, due to Alzheimer’s disease or other medical conditions causing dementia)	1	1%	60	322
Total Patients	73			

### Sensitivities and Specificities of 3D Thresholded SPECT Neuroimaging

Both pieces of data, i.e., the change in the 3D Thresholded SPECT scans and the change in the clinical functioning of the patient, are qualitative clinical interpretations, and thus at risk from bias for various reasons. However, a strong attempt was made by the radiologists to be consistent in 3D Thresholded SPECT reporting to a large extent independent of other factors. As noted above, the radiologists systematically commented on the 3D Thresholded SPECT scans, i.e., providing their opinion about whether the patient’s scan was worsening or improving, following the interpretations according to the brain SPECT atlas compiled by Amen (24).

The sensitivity, specificity, and positive predictive value for 3D Thresholded SPECT and conventional SPECT results are shown in Table 2. Analysis of before and after treatment brain 3D Thresholded SPECT scans of 73 patients with all-cause psychiatric disorders, shows that they predict improvement (i.e., non-worsening to large improvement) in clinical functioning with a sensitivity of 94.3% (95% confidence interval 86-98%) and a specificity of 66.7% (95% confidence interval 21–94%).

**TABLE 2:** Sensitivities, specificities and positive predictive values for conventional (“2D”) brain SPECT and 3D thresholded brain SPECT images in predicting improvement in psychiatric disorders.

	Sensitivity (95% confidence interval)	Specificity (95% confidence interval)	Positive predictive value (PPV) (95% confidence interval)
All Conventional (“2D”) SPECT Scans	25.7% (17-37%)	100% (44-100%)	100% (82%-100%)
All 3D Thresholded SPECT Scans	94.3% (86-98%)	66.7% (21%-94%)	98.5% (92-100%)
**Scans by diagnosis**			
Attention-Deficit Hyperactivity Disorder (ADHD)	3D:95.7% (79–99%)	3D:100% (21%–100%)	3D:100% (85–100%)
	2D:26.1% (13–47%)	2D:100% (21–100%)	2D:100% (61–100%)
Post-Mild Traumatic Brain Injury (mTBI)	3D: 89.5% (69–97%)	3D: not computable	3D: 100% (82–100%)
	2D:21% (9–43%)	2D: not computable	2D: 100% (51–100%)
Affective Disorders	3D: 100% (74–100%)	3D: not computable	3D: 100% (74–100%)
	2D:27.3% (10–57%)	2D: not computable	2D:100% (44–100%)
Psychotic Disorders	3D: 75% (30–95%)	3D: not computable	3D: 100% (44–100%)
	2D:0% (0–49%)	2D: not computable	2D: not computable
Post-Viral Chronic Syndromes	3D: 100% (44–100%)	3D: 100% (21–100%)	3D: 100% (44–100%)
	2D: 33.3% (6–79%)	2D: 100% (21–100%)	2D: 100% (21–100%)
Other Neurological	3D: 100% (44–100%)	3D: 0% (0–80%)	3D: 75% (30–95%)
	2D: 33.3% (6–79%)	2D:100% (21–100%)	2D: 100% (21–100%)

Table 2 also shows the sensitivities, specificities, and positive predictive values for 3D Thresholded SPECT scans (and as well for the conventional (“2D”) SPECT scans) in predicting improvement in the groups of patients with psychiatric disorders that occurred more commonly in this study. This is discussed in more detail below.

As noted above, due to supply issues out of the control of the clinician, 27% of the patients received Tc99m-ethyl cysteinate dimer (ECD) as the radiotracer, while the other 73% received Tc99m-HMPAO. Both Tc99m-HMPAO and Tc99m-ECD radiotracers are taken up similarly by the brain, although they have small differences in retention and imaging activity (20). For patients receiving ECD (*n* = 19), the before and after 3D Thresholded SPECT scans predicted improvements with a sensitivity of 88% (95% confidence interval 64–97%) and a specificity of 67% (95% confidence interval 21–94%). For patients receiving HMPAO (*n* = 53), the before and after 3D Thresholded SPECT scans predicted improvements with a sensitivity of 96% (95% confidence interval 87–99%). The specificity in this latter group is not computable as there are no false positives or true negatives in this sample.

### Sensitivities and Specificities of Conventional SPECT Neuroimaging

As mentioned above, for each patient the raw brain SPECT data were used to generate conventional SPECT images as well as the 3D Thresholded SPECT images. A retrospective analysis of the before and the after treatment, conventional (“2D”) brain SPECT images of the same 73 patients with all-cause psychiatric disorders, shows that they predict improvement in clinical functioning with a sensitivity of 25.7% (95% confidence interval 17–37%) and a specificity of 100% (albeit with a 95% confidence interval 44–100%). As noted above, due to the lack of full reporting by the nuclear radiologists on the conventional SPECT scans, the results are not directly comparable, although the magnitude of the values give a qualitative idea of the differences in the results of the methodologies. These values are in keeping with the low sensitivity rates found in the conventional brain SPECT scans by Schneider et al. (5) compared to the 3D Thresholded brain SPECT scans.

### Inter-Rater Reliability of Radiologists

As noted above, there were four different nuclear medicine radiologists who read different patients’ images, but only one radiologist read one patient’s images, i.e., there is no comparison possible between two different radiologists for any one particular patient’s images. However, as a rough estimate, if the patients with the largest common diagnosis in the study, which was ADHD, are examined, it is possible to see the sensitivities and specificities arrived at with different radiologists. There were three different radiologists who read the scans for patients with a diagnosis of ADHD. In Table 3 it can be seen for the two radiologists who read the vast majority of the SPECT scans of patients in this sample with ADHD, both radiologists’ scans were associated with similar sensitivities for the 3D Thresholded SPECT brain scan. Some of the specificities were not computable in Table 3 due to the true negatives and false positives both being zero in these small sample sizes.

**TABLE 3:** Sensitivities and specificities for conventional (“2D”) brain SPECT and 3D thresholded brain SPECT scans in predicting improvement in patients with a diagnosis of ADHD associated with different nuclear medicine radiologists reading the scans.

Radiologist	3D thresholded SPECT sensitivity (95% confidence limits)	3D thresholded SPECT specificity (95% confidence limits)	2D conventional SPECT sensitivity (95% confidence limits)	2D conventional SPECT specificity (95% confidence limits)
A (*n* = 7)	100% (61–100%)	100% (21–100%)	0% (0–39%)	100% (21–100%)
B (*n* = 14)	100% (79–100%)	Not computable	43% (21–67%)	Not computable
C (*n* = 3)	67% (21–94%)	Not computable	0% (0–56%)	Not computable

### Applicability of the 3D Thresholded SPECT Scan Results to All-Cause Psychiatric Disorders

As can be seen from Table 1, the most frequent diagnoses or diagnostic clusters in this study were patients with ADHD, post-mild TBI (post-mTBI), affective disorders, psychotic disorders, post-viral chronic syndromes, and a group with “other neurological” diagnoses. Table 2 shows the sensitivities, specificities, and positive predictive values for both conventional (“2D”) brain SPECT images and 3D Thresholded SPECT images in predicting improvement in the groups of patients with these psychiatric disorders. Note that these do not necessarily represent the most common diagnoses in this clinician’s practice; rather, these diagnoses were found among the complex or treatment-unresponsive cases.

## Discussion

The need exists in the community for psychiatric practitioners to be able to more objectively evaluate the response to treatment of patients with chronic psychiatric disorders. The implementation of longitudinal brain SPECT imaging in a community psychiatric practice is shown here to assist with such clinical evaluations. In considering 73 patients who received before and after treatment brain SPECT with 3D images, the SPECT scans predicted clinical improvement (non-worsening to large improvement) with a sensitivity of 94% (95% confidence interval 86–98%) and a specificity of 67% (95% confidence interval 21–94%). Thus, 3D thresholded brain SPECT imaging can assist the community psychiatric practitioner in the management of patients.

The more conventional 2D brain SPECT images from the same patients unfortunately predicted clinical improvement (non-worsening to large improvement) with a sensitivity of only 26% (95% confidence interval 17–37%). Thus, the data from this community study would indicate that the more conventional 2D brain SPECT imaging is less useful in assisting the community psychiatric practitioner in the management of patients. Other types of neuroimaging would also be expected to be less useful to the community psychiatric practitioner. For example, Raji et al. (28) review the literature to compare SPECT neuroimaging with other forms of neuroimaging in the setting of TBI. In this setting, conventional brain SPECT, as poor as it performed in our study above, was found to have significant advantages compared to computerized tomography (CT) or MRI in the detection of acute and chronic TBI, especially in mild TBI.

Although the sensitivity of the conventional 2D brain SPECT is poor at 26% (95% confidence interval 17–37%) its specificity was 100% (albeit with a 95% confidence interval 44–100%). If the confidence limits of the specificity are disregarded for a moment, the higher specificity of the conventional 2D brain SPECT is expected as changes in the brain images are more apparent on 3D images rather than on 2D images, and so, there will be fewer false positives read in the 2D conventional group (i.e., small changes will not appear significant as they may in a 3D thresholded image and will almost always be read as negative in the 2D image). In fact, in the 73 patients considered in this study, there were no false positives at all in the 2D brain SPECT results. Indeed, the specificity of the 3D thresholded brain SPECT was found to be lower at 67% (95% confidence interval 21–94%). On the other hand, the sensitivity of the 3D thresholded brain SPECT was found to be much higher at 94% (95% confidence interval 86–98%) again reflecting the ability to detect changes easier in the 3D thresholded brain SPECT scan.

From the perspective of the community psychiatric practitioner there is the question of whether a potential objective test such as brain SPECT imaging is applicable to only one particular diagnosis, [e.g. TBI, see (28)], or whether it can be useful in a community practice consisting of patients with a variety of psychiatric disorders. Even with regard to applying SPECT scanning to a particular diagnosis, Henderson and colleagues (29, 30), note that psychiatric diagnoses as defined by the Diagnostic and Statistical Manual 5 (DSM-5) of the American Psychiatric Association (APA) lack distinct boundaries from other diagnoses, as well as covering multiple combinations of disparate symptoms within the confines of a single diagnosis. Thus, it becomes difficult to identify a pure cohort of subjects with a common set of symptoms fitting a single distinct DSM-5 diagnosis in order to test a potential biomarker for sensitivity and specificity. As is shown in Table 1, the 73 patients studied in the community practice represented a variety of psychiatric diagnoses or diagnostic clusters. The most frequent diagnoses or diagnostic clusters in this study were patients with ADHD, post-mild TBI (post-mTBI), affective disorders, psychotic disorders, post-viral chronic syndromes and a group with “other neurological” diagnoses that were referred to psychiatry. Table 2 shows the sensitivities, specificities, and positive predictive values for 3D Thresholded SPECT scans in predicting improvement in the groups of patients with these psychiatric disorders. Additional research is required in order to evaluate the utility of 3D Thresholded SPECT scans in the large variety of patients the community psychiatric practitioner can encounter, but the results in Table 2 indicate that it would seem by logical induction reasonable to consider that 3D Thresholded SPECT scans would yield useful results in the real-world diverse cases that present to the community psychiatric practice.

## Data Availability Statement

The datasets presented in this article are not readily available because they are derived from clinical patient data. Prefer to keep as confidential as possible. Requests to access the datasets should be directed to JT, jfthor@hotmail.com.

## Ethics Statement

Ethical review and approval was not required for the study on human participants in accordance with the local legislation and institutional requirements. Written informed consent for participation was not required for this study in accordance with the national legislation and the institutional requirements.

## Author Contributions

JT was involved in the clinical care and imaging of patients, organizing, drafting, writing, and editing the manuscript. HS and TH were involved in organizing, drafting, writing, and editing the manuscript, and discussions concerning patient care and imaging. PC, MM, ML, SD, Y-HS, and JU were involved in organizing and editing the manuscript, and discussions concerning patient care and imaging. DP deceased at time of submission, was involved in discussions concerning patient care and imaging. All authors contributed to the article and approved the submitted version.

## Conflict of Interest

SD is President at Good Lion Imaging LLC, a neuroimaging-related company. JU is the Medical Director of DrSpectScan.org, a clinical service corporation and derives 25% of his income from neuroimaging. Y-HS derives 10% of his income from neuroimaging. DP is deceased. TH is the president and principal owner of The Synaptic Space, a neuroimaging consulting firm. He is also CEO and Chairman of the Board of Neuro-Luminance Corporation, a medical service company. He is also president and principal owner of Dr. Theodore Henderson, Inc, a medical service company. TH has no ownership in, and receives no remuneration from, any neuroimaging company. No more than 5% of his income is derived from neuroimaging. The remaining authors declare that the research was conducted in the absence of any commercial or financial relationships that could be construed as a potential conflict of interest.

## Publisher’s Note

All claims expressed in this article are solely those of the authors and do not necessarily represent those of their affiliated organizations, or those of the publisher, the editors and the reviewers. Any product that may be evaluated in this article, or claim that may be made by its manufacturer, is not guaranteed or endorsed by the publisher.
